# Neutrophils are dispensable in the modulation of T cell immunity against cutaneous HSV-1 infection

**DOI:** 10.1038/srep41091

**Published:** 2017-01-23

**Authors:** Jyh Liang Hor, William R. Heath, Scott N. Mueller

**Affiliations:** 1 Department of Microbiology and Immunology, The University of Melbourne, at the Peter Doherty Institute for Infection and Immunity, Melbourne 3000, Australia; 2 The Australian Research Council Centre of Excellence in Advanced Molecular Imaging, The University of Melbourne, Melbourne 3000, Australia

## Abstract

Neutrophils rapidly infiltrate sites of inflammation during peripheral infection or tissue injury. In addition to their well described roles as pro-inflammatory phagocytes responsible for pathogen clearance, recent studies have demonstrated a broader functional repertoire including mediating crosstalk between innate and adaptive arms of the immune system. Specifically, neutrophils have been proposed to mediate antigen transport to lymph nodes (LN) to modulate T cell priming and to influence T cell migration to infected tissues. Using a mouse model of cutaneous herpes simplex virus type 1 (HSV-1) infection we explored potential contributions of neutrophils toward anti-viral immunity. While a transient, early influx of neutrophils was triggered by dermal scarification, we did not detect migration of neutrophils from the skin to LN. Furthermore, despite recruitment of neutrophils into LN from the blood, priming and expansion of CD4^+^ and CD8^+^ T cells was unaffected following neutrophil depletion. Finally, we found that neutrophils were dispensable for the migration of effector T cells into infected skin. Our study suggests that the immunomodulatory roles of neutrophils toward adaptive immunity may be context-dependent, and are likely determined by the type of pathogen and anatomical site of infection.

Innate and adaptive immunity form two distinct arms of the immune system, but recent research increasingly points to a close cooperation between immune cells of both arms. Neutrophils are the principal innate immune cells to rapidly infiltrate peripheral tissues in response to infection and tissue damage. Sensing of pathogen-associated molecular patterns (PAMP) or danger-associated molecular patterns (DAMP) released by infected or damaged cells leads to secretion of inflammatory factors by tissue-resident macrophages and mast cells, triggering neutrophil recruitment to the foci of inflammation^1^,^2^.

Neutrophils have long been established as terminally differentiated cells responsible for the inflammatory response as well as pathogen clearance through phagocytosis and degranulation of reactive oxygen species. More recently, neutrophils were reported to perform immunomodulatory functions and engage in crosstalk between the innate and adaptive branches of the immune system^3^. In particular, neutrophils have been implicated in transporting bacterial antigens to draining LN following *M. bovis* BCG inoculation of the ear pinna^4^, and their migration to the LN from the skin after *S. aureus* injection may influence humoral and cell-mediated responses^5^. This raises the possibility that their numerical advantage and rapid infiltration into infection sites could provide prompt delivery of antigen to the lymphoid tissues and enhance antigen presentation, especially when the initial antigen load is low. Others have also found that neutrophils interact with B cells and plasma cells in the LN to regulate humoral immunity^6^. More recently, neutrophils were also shown to facilitate migration of effector CD8^+^ T cells into influenza-infected trachea via deposition of chemokine trails^7^. Thus, there is mounting evidence to suggest the contribution of neutrophils in shaping the adaptive immune system.

Studies investigating the role of neutrophils during peripheral viral infections, including influenza virus in the lung, and herpes simplex virus type 1 (HSV-1) in the skin, have reported their recruitment to sites of infection at the peak of infection, but have not examined their role early in the initiation of adaptive immunity^8^,^9^. Functional studies showed that neutrophils enhanced CD8^+^ T cell responses against influenza virus in the lung^2^,^8^, but had no effect on the viral load nor the severity of disease during both intranasal and epicutaneous HSV-1 infections^9^,^10^. The relationship between neutrophils and priming of T cells during skin HSV-1 infection, however, was not assessed.

Here, we investigated whether neutrophils: (1) help enhance antigen transport to the draining LN, and (2) contribute to the priming, expansion and migration of CD4^+^ and CD8^+^ T cells during cutaneous HSV-1 infection. We show that while neutrophils infiltrate both the skin and draining LN early after dermal scarification, skin-infiltrating neutrophils do not migrate to draining LN. These cells are dispensable for the priming and expansion of primary T cell responses against HSV-1, and are not required for the migration of effector cells into infected skin. Our results argue that contributions by neutrophils in shaping adaptive immunity may be dependent upon the specific context of infection, including the type of pathogen and the anatomical site of infection.

## Results

### Early virus-independent recruitment of neutrophils to HSV-infected skin

**Figure 1:**
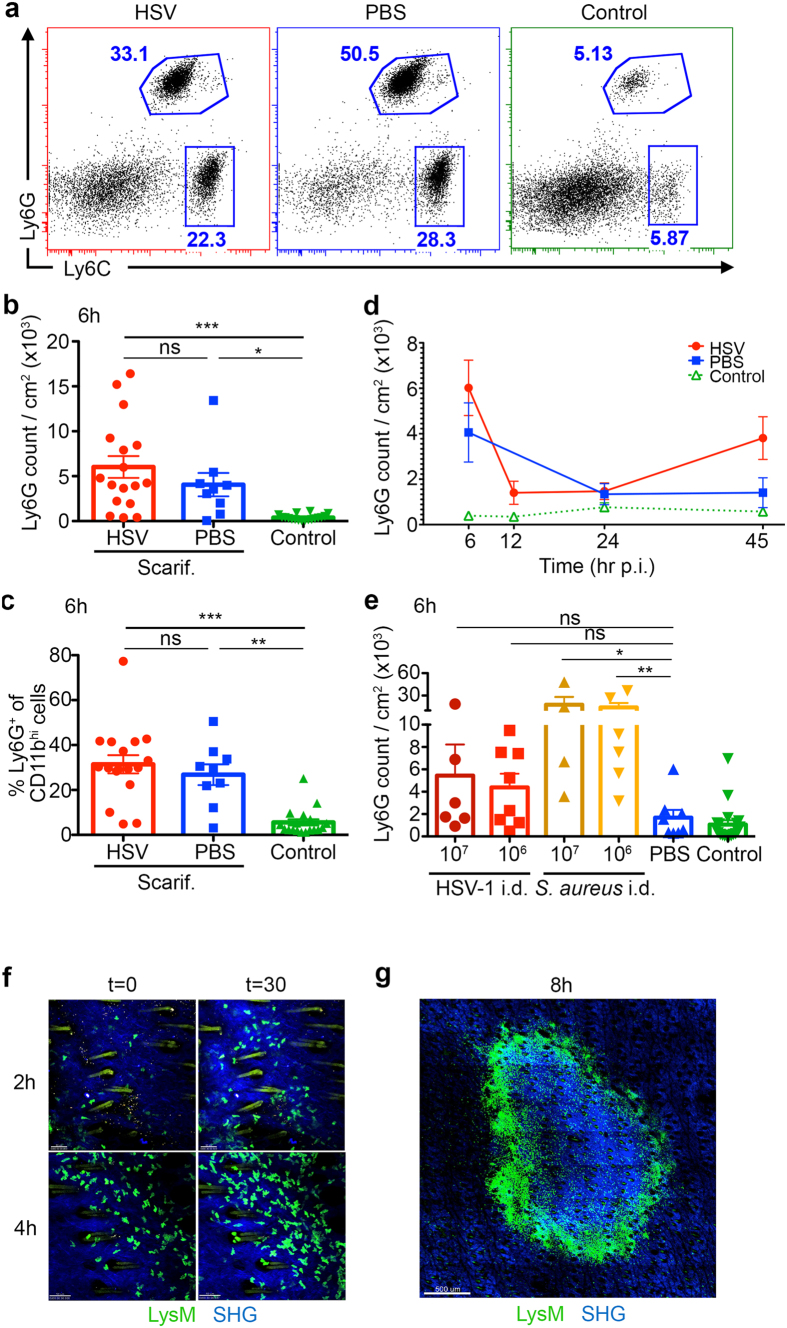
Early virus-independent recruitment of neutrophils to HSV-infected skin. (**a**) C57BL/6 mice were scarified and treated with either HSV-1 (red box), PBS (blue box) or left unscarified (green box). 1 cm^2^ skin was excised for flow cytometric analysis at various time points. Representative dot plots of CD11b^+^ cells in the skin at 6 hr post-scarification are shown in (**a**), also depicting gating strategies for neutrophils (Ly6G^hi^ Ly6C^int^) and monocytes (Ly6C^hi^ Ly6G^lo^). (**b**) Total numbers of Ly6G^hi^ neutrophils recovered from skin at 6 hr post-scarification, as shown in (**a**). (**c**) Proportion of Ly6G^hi^ neutrophils amongst CD11b^+^ population at 6 hr post-scarification, as shown in (**a**). (**d**) Time course depicting total number of Ly6G^hi^ neutrophils in skin from 6 hr to 45 hr post-scarification. (**a**–**d**) Data pooled from 2–5 independent experiments, n = 9–18 mice per group (**b**,**c**), n = 5–19 mice per group (**d**). (**e**) C57BL/6 mice were intradermally injected with HSV-1 (10^7 ^pfu, dark red or 10^6 ^pfu, red), *S. aureus* (10^7 ^cfu, dark orange or 10^6 ^cfu, orange), PBS (blue) or left untreated (green). Skin containing the injection site was excised at 6 hr p.i. for flow cytometry. Shown are total numbers of Ly6G^hi^ neutrophils recovered from 1 cm^2^ skin. Data pooled from 2 independent experiments, n = 4–8 per group, n = 19 for control group. (**a**–**e**) Error bars represent mean ± SEM. *p < 0.05, **p < 0.01, ***p < 0.001, by one-way ANOVA. ns, not significant. (**f**,**g**) LysM-EGFP mice expressing fluorescent neutrophils (green) were scarified and inoculated with HSV-1 at least 1 hr before intravital two-photon microscopy. (**f**) Time lapse snapshots depicting two-areas of scarified skin (2 hr, top row, and 4 hr, bottom row after HSV-1 infection) at the start of the imaging period (t = 0) and 30 minutes after (t = 30). Neutrophils are shown in green amongst dermal collagen (blue, SHG). Macrophages and monocytes are visible as dim green fluorescent cells. Scale bars denote 50 μm. See also Supplementary Video S1. (**g**) Snapshot of the entire scarified region at 8 hr after HSV-1 infection, showing localisation of neutrophils (bright green) in relation to the scarified area. Scale bar denotes 500 μm.

Neutrophils infiltrate the skin after epicutaneous HSV-1 infection with their numbers peaking 5 days after the initial infection^9^. However, the kinetics of their influx into the skin and draining LNs during the early hours after infection remained unclear. Using flow cytometry to follow the recruitment of neutrophils into the skin, we observed significant influx of Ly6G^hi^ Ly6C^int^ neutrophils as early as 6 hr p.i. (Fig. 1a) In addition, Ly6C^hi^ monocytes also showed an increase in their numbers (Fig. 1a, Supplementary Fig. S1a).

Although there was variation in the number of neutrophils between mice, there was significant increase in this cell population in infected skin compared to control skin (Fig. 1b), with neutrophils representing ~30% of total CD11b^+^ myeloid cells in infected skin (Fig. 1c). A comparable accumulation of neutrophils in mock-infected (PBS-treated) skin, however, suggested that recruitment was independent of viral-derived cues. A similar trend was observed for Ly6C^hi^ monocytes (Supplementary Fig. S1a,b,c).

Following the kinetics of neutrophil recruitment to the skin over the first 45 hr of infection revealed this early influx of granulocytes at 6 hr p.i. represented a transient wave of recruitment, with a decline in numbers by 12 hr p.i., reaching near baseline levels at around 24 hr p.i. before increasing again at 45 hr p.i. (Fig. 1d) This secondary wave of recruitment, which was infection specific, is expected to peak at around Day 5 p.i. at a higher magnitude than the earlier peak at 6 hr p.i., as has been reported previously^9^.

### Minor contribution of viral-derived cues in neutrophil recruitment to the skin

While neutrophils clearly infiltrated the skin even in the absence of virus, it was not clear if virus-induced inflammation also contributed to their recruitment, which might have been obscured by inflammation triggered by scarification. To investigate if viral-derived factors contribute to their recruitment to the skin, we injected mice with either HSV-1 (a normal dose of 10^6 ^pfu, or a high dose of 10^7 ^pfu) or saline intradermally and analysed the skin at 6 hr p.i. (Fig. 1e). Intradermal injection creates minimal tissue damage compared to scarification, while delivery of virus into the dermis prevents epidermal lesions due to the epidermal tropism of HSV.

As a control, we also compared the extent of neutrophil infiltration into the skin with a bacterial pathogen. It is well documented that *S. aureus* infection of ear skin induces robust neutrophil recruitment to the injection site^11^. We postulated that intradermal lodgment of this pathogen in flank skin would also induce similar levels of neutrophil recruitment.

We observed that while injection of either HSV-1 or *S. aureus* led to increased neutrophil cellularity in the skin, the increase with HSV-1 was not significantly greater than the PBS control (Fig. 1e). Similarly, there was a trend towards increased monocyte accumulation under both infectious conditions but these differences were not significant (Supplementary Fig. S1d). In summary, our data indicates that there may be minor contribution of viral-derived factors to neutrophil recruitment in the skin, though the majority of their influx into scarified and infected skin is likely due to tissue damage caused by scarification.

### Neutrophils localised near infected keratinocytes during early skin infiltration

We next sought to determine the localisation of skin-infiltrating neutrophils via intravital two-photon microscopy. To visualise neutrophil migration in the skin, we used transgenic LysM-EGFP mice, where neutrophils and monocytes/macrophages express enhanced green fluorescent protein (EGFP), and can be distinguished through their respective bright or dim expression of the fluorescent protein and the high motility of the neutrophils.

As early as 2 hr after infection, neutrophils entered the dermis of the scarified site, localising predominantly to the edge of the scarified region, where exposed epidermal keratinocytes were infected (Fig. 1f and Supplementary Video S1). Accumulation of neutrophils at the scarified site continued over the next few hours, and by 8 hr p.i., the entire perimeter of the scar was illuminated by bright aggregates of EGFP^+^ neutrophils (Fig. 1g). Our intravital imaging data thus demonstrated that the positioning of these early, skin-infiltrating neutrophils, near infected keratinocytes in the epidermis, presented the opportunity to interact with infected cells and possibly to acquire viral antigens.

### Concurrent infiltration of neutrophils into draining LN following dermal scarification

**Figure 2:**
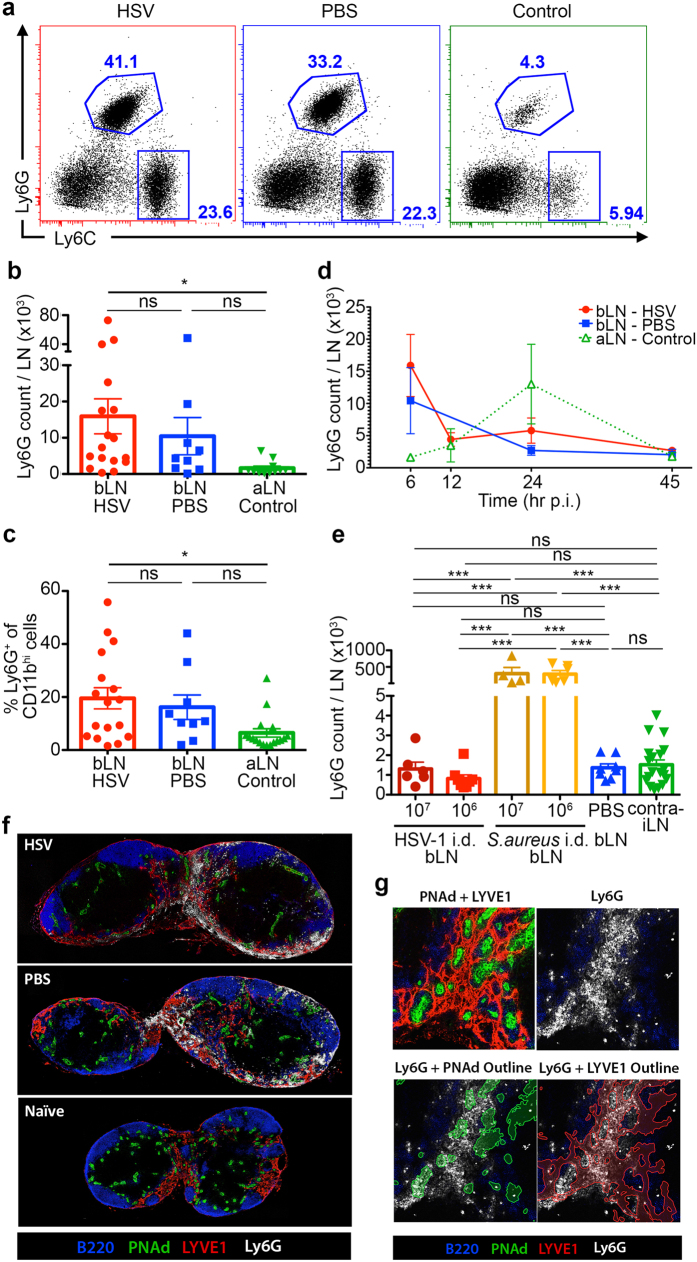
Concurrent infiltration of neutrophils into draining LN following dermal scarification. (**a**) Draining bLNs corresponding to Fig. 1(a–d) were analysed by flow cytometry. Representative dot plots of CD11b^+^ cells in bLN draining the skin treated with HSV-1 (red box), PBS (blue box), or left untreated (green box). Also shown are gating strategies for neutrophils (Ly6G^hi^ Ly6C^int^) and monocytes (Ly6C^hi^ Ly6G^lo^). (**b**) Total number of Ly6G^hi^ neutrophils recovered from bLN at 6 hr post-scarification, as shown in (**a**). (**c**) Proportion of Ly6G^hi^ neutrophils amongst CD11b^+^ population at 6 hr post-scarification, as shown in (**a**). (**d**) Time course depicting total numbers of Ly6G^hi^ neutrophils 6 hr to 45 hr post-scarification. (**a**–**d**) Data pooled from 2–5 independent experiments, n = 9–18 mice per group (**b**,**c**), n = 5–18 mice per group (**d**). (**e**) Draining bLNs corresponding to Fig. 1e intradermally injected with HSV-1 (10^7 ^pfu, dark red or 10^6 ^pfu, red), *S. aureus* (10^7 ^cfu, dark orange or 10^6 ^cfu, orange), PBS (blue) or left untreated (green), were harvested at 6 hr p.i. Shown are total numbers of Ly6G^hi^ neutrophils recovered from the LNs. Data pooled from 2 independent experiments, n = 4–8 per group, n = 19 for control group. (**a**–**e**) Error bars represent mean ± SEM. *p < 0.05, **p < 0.01, ***p < 0.001, one-way ANOVA, Tukey’s multiple comparisons. ns, not significant. (**f**,**g**) C57BL/6 mice were scarified in the flank skin and treated with either HSV-1 or PBS. Draining bLNs were harvested at 6 hr p.i. (**f**) Confocal images of bLN sections stained with anti-B220 (blue), anti-PNAd (green), anti-LYVE1 (red) delineating B cells, HEVs, and lymphatic vessels respectively. Localisation of neutrophils was visualised using anti-Ly6G (white). (**g**) Close-up of medullary region of bLN from HSV-1 infected mouse (top) showing location of neutrophils (white) in both HEVs (green) and lymphatic sinuses (red). (**f**,**g**) Data representative of 2 independent experiments, n = 4–6 mice per group.

In addition to the skin, we also examined the draining brachial LNs (bLN) and noted an influx of neutrophils and monocytes into the LN concurrent with those in the skin (Fig. 2a). Neutrophils and monocytes recovered from the LN were in greater numbers at 6 hr p.i. compared to those enumerated in the corresponding skin samples (Fig. 2b, Supplementary Fig. S2a), and similar to the skin, represented ~15–20% of total CD11b^+^ myeloid cells in bLN (Fig. 2c, Supplementary Fig. S2b). Saline-treated mice showed slightly fewer neutrophils but this difference was not significant. Paralleling the skin, their numbers began to decline by 12 hr p.i. and returned to baseline by 45 hr p.i. (Fig. 2d) Although the downstream axillary LN (aLN), which we used as controls, showed an elevated neutrophil response at 24 hr p.i., this was largely caused by two outliers while the rest showed similar levels of neutrophil number compared to baseline.

Intradermal injection of HSV-1 failed to show any increase in neutrophils in the draining bLN (Fig. 2e). This contrasted with injection of *S. aureus*, which yielded high numbers of neutrophil in the bLN (Fig. 2e). Monocyte numbers were highly variable between individual experiments, although the reason for this is unknown (Supplementary Fig. S2d). These findings suggest that for HSV infection, only dermal scarification caused an influx of neutrophils in the LN, while viral drainage was largely irrelevant to neutrophil recruitment into the LN.

Nonetheless, the simultaneous influx of neutrophils into the skin and LN after HSV infection of scarified skin suggested either an independent recruitment of the cells to both tissues concurrently, or a continuity where neutrophils first entered the skin, from which they then migrated into the draining bLN.

To explore if there was a connection between neutrophils in the skin and LN, we performed immunofluorescence microscopy on bLN sections at 6 hr p.i., at the peak of neutrophil migration into the LN. By staining for high endothelial venules (HEVs) and lymphatic vessels, we wished to determine the localisation of neutrophils in the bLN: neutrophils situated in the subcapsular sinus (SCS) could indicate entry from afferent lymphatics, possibly from the peripheral tissues; in contrast, neutrophils from the bloodstream would enter from HEVs.

We found that most neutrophils distributed to the medullary regions of bLN in both virus- and PBS-treated mice, where lymphatic sinuses are abundant and many of which intertwined with HEVs in the medullary region (Fig. 2f). Many neutrophils were situated within the medullary lymphatic sinuses as well as around and within HEVs (Fig. 2f,g). Interestingly, neutrophil localisation occurred almost exclusively around medullary HEVs, rather than paracortical HEVs. Although we observed few neutrophils in the SCS, based on these confocal microscopy data we could not determine if the neutrophils entered the LN via HEVs or afferent lymphatics. Very few neutrophils were found within T or B cell zones at the time point examined, which suggested that there were probably minimal interactions with T and B cells.

### Neutrophils do not migrate from the skin to dLN after HSV infection

Although neutrophils and monocytes infiltrating the draining LN after epicutaneous HSV infection might be recruited independently of those infiltrating the skin, the localisation of neutrophils to the SCS and medullary sinuses of the dLN suggested that some could have migrated from the skin. We therefore examined if this was the case and determined whether skin-to-LN migrant cells could potentially serve as carriers of virus or viral antigens to the dLN.

**Figure 3:**
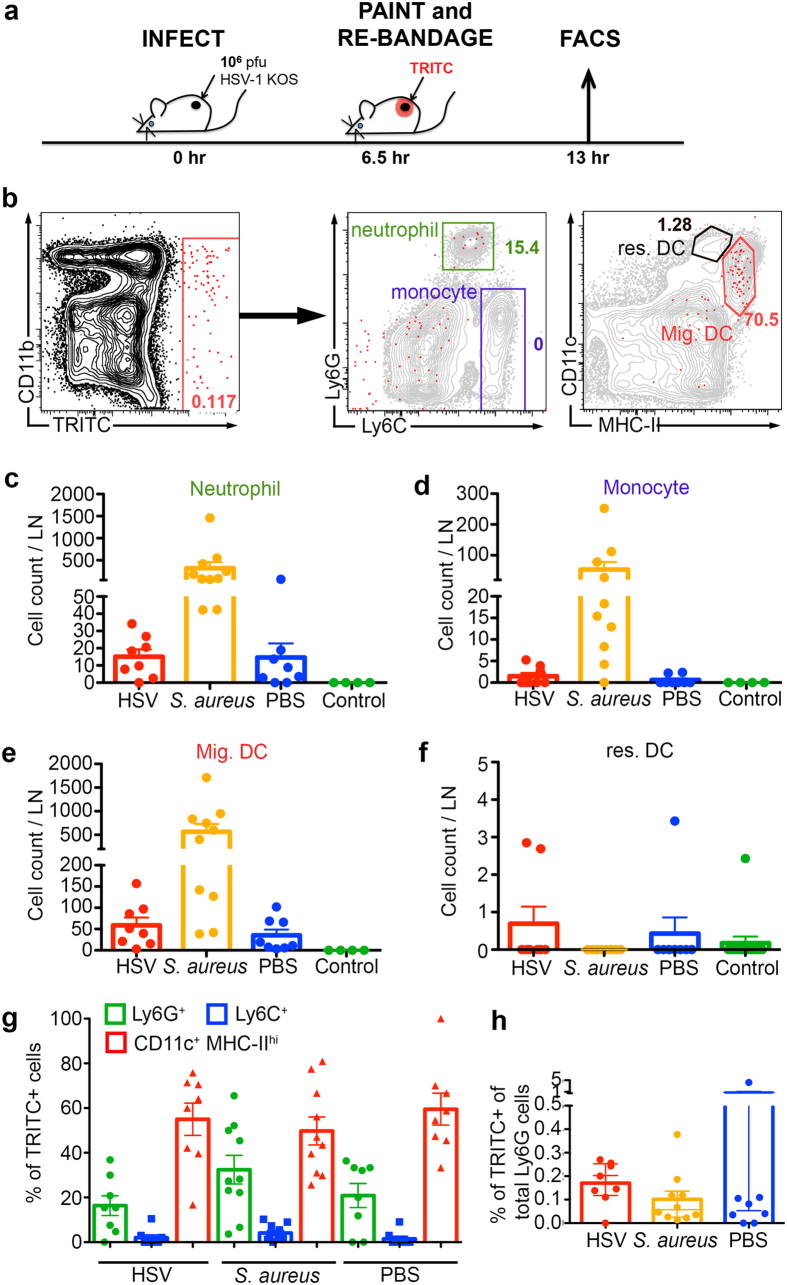
Minimal migration of neutrophils from the skin to dLN after HSV infection. (**a**) Schematic showing the procedure for tracking neutrophil migration from skin to bLN. C57BL/6 mice were scarified and treated with either 10^6 ^pfu HSV-1 or PBS, or i.d. injection with 10^6 ^CFU *S. aureus*. Mice were painted with fluorescent dye TRITC and re-bandaged at 6.5 hr post-scarification prior to sacrifice at 13 hr post-scarification. (**b**) Contour plots showing the gating strategy for TRITC^+^ cells in draining bLN. TRITC^+^ cells were further separated into CD11c^+^ MHC-II^hi^ migratory dDC (red), CD11c^+^ MHC^int^ LN-resident DC (black), Ly6C^hi^ monocytes (blue), and Ly6G^hi^ neutrophils (green). (**c**–**f**) Total numbers of TRITC^+^ Ly6G^hi^ neutrophils (**c**), Ly6C^hi^ monocytes (**d**), CD11c^+^ MHC-II^hi^ migratory dDC (**e**), and CD11c^+^ MHC-II^int^ LN-resident DC (**f**) in bLN as gated in (**b**) under various conditions. (**g**) Proportion of TRITC^+^ Ly6G^hi^ neutrophils (green), Ly6C^hi^ monocytes (blue), and CD11c^+^ MHC-II^hi^ migratory dDC (red) amongst all TRITC^+^ cells in bLN of either HSV-1-, *S. aureus*- or PBS-treated mice at 13 hr p.i. (**h**) Proportion of TRITC^+^ cells of total Ly6G^hi^ neutrophils per bLN of either HSV-1- (red), S. aureus- (yellow) or PBS-treated mice (blue) at 13 hr p.i. Data pooled from 2 independent experiments, n = 4–10 LN per group. Error bars represent mean ± SEM.

To track their migration from the skin, we painted the infection site with the red fluorescent dye TRITC at ~6 hr p.i., during the peak recruitment of neutrophils and monocytes to the skin, and analysed the dLN at 13 hr p.i. after allowing sufficient time for migration (Fig. 3a). As bacterial infections can induce neutrophil migration to the dLN from the ear skin injected with killed *S. aureus* bioparticles^7^, we also examined intradermal *S. aureus* infection of the flank skin for comparison purposes.

Only very low numbers of TRITC^+^ cells were recovered in the draining bLN (less than 100 cells on average) after epicutaneous HSV or PBS treatment, and these cells were mostly comprised of migratory dermal DC (dDC) (~60%) as identified by their CD11c^+^ MHC-II^hi^ phenotype (Fig. 3b,e,g). Conversely, CD11c^+^ MHC-II^int^ LN-resident DC were not TRITC^+^ (Fig. 3b,f). This ruled out the possibility of dye capture by antigen-presenting cells (APC) in the LN via lymphatic drainage.

For neutrophils, we detected very few TRITC^+^ Ly6G^hi^ cells after HSV infection (on average less than 20 neutrophils per LN), while equivalent numbers were found in the saline-treated group (Fig. 3b,c). These migrant neutrophils contributed to ~20% of the total TRITC^+^ cells (Fig. 3g). Surprisingly, there was a near complete absence of Ly6C^hi^ monocytes migrating to dLN (Fig. 3d), in contrast to studies suggesting antigen transport by monocytes from peripheral tissues to the LN following intranasal administration of ovalbumin (OVA) or anthrax spores^12^.

By comparison, injection of 10^6 ^cfu *S. aureus* induced a higher number of migrating neutrophils, monocytes and dDC to the dLN, amounting to ~250 neutrophils, ~100 monocytes and ~500 migratory dDC per LN (Fig. 3c,d,e). Interestingly, the proportions of TRITC^+^ neutrophils and dDC amongst total TRITC^+^ cells were similar to those of HSV and saline-treated groups (Fig. 3g), indicating that the different pathogens induced a similar proportion but different magnitude of cell migration from skin to dLN. It should be noted that TRITC^+^ neutrophils represented only ~0.1–0.2% of total neutrophils in the draining bLN under all conditions tested (Fig. 3.7 h). Moreover, although ~13% of neutrophils in the skin harboured *S. aureus*, these were not observed migrating to the draining LN (Supplementary Fig. S3). Thus, after live *S. aureus* infection the majority of dLN-infiltrating neutrophils arrived from the blood and of the neutrophils that migrated from the skin, few were observed to transport bacteria to the draining LN.

Taken together, these data demonstrate minimal neutrophil migration from the skin to dLN early after cutaneous HSV infection. Although bacterial infection induced greater neutrophil migration than HSV, the absence of *S. aureus* containing cells suggests that neutrophils likely do not function as substantial antigen carriers during skin infection.

### Neutrophils are dispensable for CD4^+^ and CD8^+^ T cell priming and expansion during cutaneous HSV-1 infection

**Figure 4:**
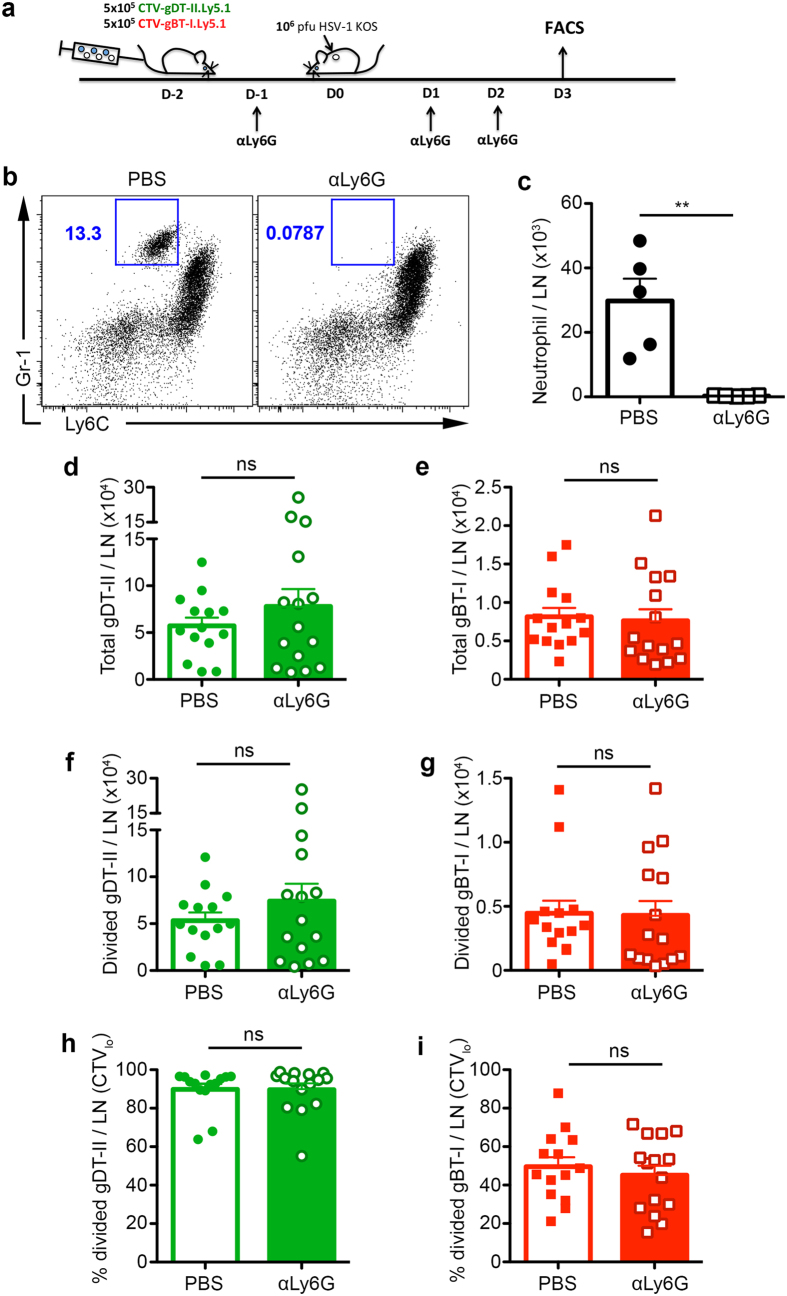
Neutrophils are dispensable for CD4^+^ and CD8^+^ T cell priming and expansion during cutaneous HSV-1 infection. (**a**) Schematic showing the strategy for neutrophil depletion. 5 × 10^5^ CellTrace Violet-labelled CD4^+^ gDT-II and CD8^+^ gBT-I cells were adoptively transferred into C57BL/6 mice 2 days before epicutaneous infection with 10^6 ^pfu HSV-1. 500 μg of 1A8 (anti-Ly6G) depleting antibodies were injected i.p. into mice from the depleted group 1 day before, and 1 and 2 days after infection. Non-depleted mice were treated with PBS. Mice were sacrificed at Day 3 p.i. for flow cytometry analysis. (**b**) Representative dot plots showing neutrophil depletion efficiency as indicated by co-staining for Ly6C and Gr-1. (**c**) Total number of neutrophils recovered from draining bLN in both non-depleted and anti-Ly6G depleted groups based on gating shown in (**b**).(**d**,**e**) Total number of CD4^+^ gDT-II (**d**) and CD8^+^ gBT-I (**e**) T cells recovered from bLN in non-depleted (open bars) and anti-Ly6G depleted (filled bars) groups. (**f**,**g**) Total number of divided (CellTrace Violet_lo_) CD4^+^ gDT-II (**f**) and CD8^+^ gBT-I (**g**) T cells recovered from bLN in non-depleted (open bars) and anti-Ly6G depleted (filled bars) groups. (**h**,**i**) Proportion of divided (CellTrace Violet_lo_) CD4^+^ gDT-II (**h**) and CD8^+^ gBT-I (**i**) T cells in non-depleted (open bars) and anti-Ly6G depleted (filled bars) groups. Data pooled from 3 independent experiments, n = 14–15 mice per group. Error bars represent mean ± SEM. **p < 0.01, unpaired Student t test (**c**,**h**,**i**) and unpaired Student t test on log transformed values (**d**–**g**). ns, not significant.

Neutrophils migrating from the skin to LN have been implicated in modulating T cell expansion during dermal *S. aureus* infection^5^. To investigate whether neutrophils play vital roles in T cell priming and proliferation, we co-transferred CellTrace Violet-labelled HSV gD-specific CD4^+^ gDT-II and gB-specific CD8^+^ gBT-I T cells into recipient mice, then depleted neutrophils from the mice by administering 3 doses of anti-Ly6G antibodies at Days -1, 1 and 2 p.i. Draining bLNs were examined at 72 hr following epicutaneous HSV-1 infection to assess expression of activation markers as well as cell division as measured by CellTrace Violet dilution (Fig. 4a).

To determine depletion efficiency, we co-stained bLN cells with antibodies against Ly6C and Gr-1 to detect neutrophils and found almost complete ablation in the treated group (Fig. 4b,c). However, the resultant expansion of both CD4^+^ and CD8^+^ T cells (Fig. 4d,e,f,g), as well as their extent of division (Fig. 4h,i), was comparable between groups treated with depleting antibodies or left untreated. This suggested that neutrophils did not markedly affect T cell expansion. Although there was a higher CD4^+^ T cell count compared to CD8^+^ T cells, this was attributed to the early priming of CD4^+^ T cells during cutaneous HSV-1 infection^13^. Furthermore, expression of cell surface markers associated with T cell activation: CD69, CD62L and CD44 were similar between both groups (Supplementary Fig. S4). Together, these results support the view that neutrophils are dispensable for T cell priming and early proliferation after skin HSV-1 infection.

### Neutrophils do not contribute to effector CD4^+^ and CD8^+^ T cell homing to the skin after HSV-1 infection

**Figure 5:**
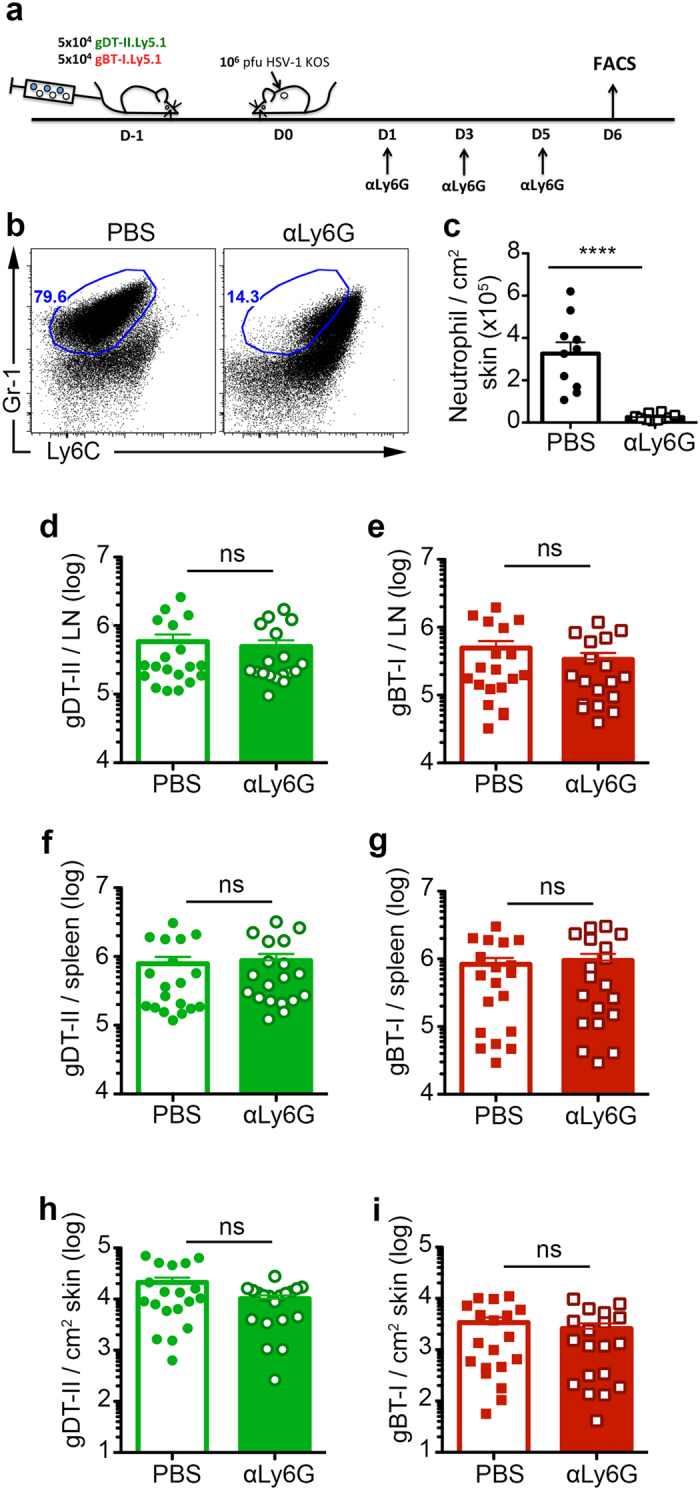
Neutrophils do not contribute to effector CD4^+^ and CD8^+^ T cell homing to the skin after HSV-1 infection. (**a**) Schematic showing neutrophil depletion strategy. 5 × 10^4^ CD4 gDT-II and CD8 gBT-I T cells each were adoptively transferred into C57BL/6 mice prior to epicutaneous HSV-1 infection. Mice received 3 doses of 500 μg 1A8 (anti-Ly6G) depleting antibodies i.p. at Days 1, 3 and 5 p.i. and were sacrificed at Day 6 p.i. Skin, draining bLN and spleens were harvested for analysis. Non-depleted mice were treated with PBS. (**b**) Representative dot plots showing neutrophil depletion efficiency in the skin as indicated by co-staining for Ly6C and Gr-1. (**c**) Numbers of neutrophils in skin of both non-depleted and anti-Ly6G depleted groups based on gating shown in (**b**). (**d**,**e**) Total number of CD4^+^ gDT-II (**d**) and CD8^+^ gBT-I (**e**) T cells recovered from Day 6 p.i. bLN in non-depleted (open bars) and anti-Ly6G depleted (filled bars) groups. (**f**,**g**) Total number of CD4^+^ gDT-II (**f**) and CD8^+^ gBT-I (**g**) T cells recovered from Day 6 p.i. spleen in non-depleted (open bars) and anti-Ly6G depleted (filled bars) groups. (**h**,**i**) Total number of CD4^+^ gDT-II (**h**) and CD8^+^ gBT-I (**i**) T cells per cm^2^ of excised Day 6 p.i. skin in non-depleted (open bars) and anti-Ly6G depleted (filled bars) groups. Data pooled from 4 independent experiment, n = 18–19 mice per group. Error bars represent mean ± SEM. ****p < 0.001, unpaired Student t test (**c**) and unpaired Student t test on log transformed values (**e**–**i**). ns, not significant.

Neutrophils have also been demonstrated to influence CD8^+^ T cell migration into influenza-infected trachea via deposition of CXCL12 chemokine trails from blood vessels to the infected region^7^. We wanted to investigate if neutrophils are also crucial for the homing of effector T cells into skin during HSV-1 infection. To compare effector cell numbers in the skin, we co-transferred 5 × 10^4^ congenically-marked HSV-specific CD4^+^ gDT-II and CD8^+^ gBT-I T cells into recipient mice before infection, administered anti-Ly6G depleting antibodies at Days 1, 3 and 5 p.i., prior to analysing the infected skin at Day 6 p.i. via flow cytometry (Fig. 5a). Although an increase in Ly6C^int^ Gr-1^int^ cells in anti-Ly6G-treated mice caused some difficulty in gating on neutrophils in the skin, possibly due to reduced Gr-1 staining, their numbers appeared to be depleted to less than a tenth of control mice after treatment with anti-Ly6G mAb (Fig. 5b,c).

Examining the lymphoid tissues, we found similar numbers of CD4^+^ gDT-II and CD8^+^ gBT-I T cells in the spleen and draining bLN on Day 6 p.i. between both neutrophil-depleted and control groups (Fig. 5d,e,f,g). This indicated that, as shown in the previous section, HSV-specific T cell expansion was unaffected by the ablation of neutrophils. Next, we enumerated CD4^+^ gDT-II and CD8^+^ gBT-I T cells in the skin 6 days after infection. From four independent experiments, we noted mostly equivalent numbers in both the neutrophil-depleted and control groups of mice (Fig. 5h,i). This was also similar when the skins were examined at 7 days after infection (Supplementary Fig. S5a,b). Additionally, endogenous CD4^+^ and CD8^+^ T cells also showed similar numbers in the skin, bLN and spleen at Day 6 p.i. (Supplementary Fig. S4c–h) Together, our results show that neutrophils do not contribute to effector CD4^+^ and CD8^+^ T cell recruitment to the skin after HSV infection.

## Discussion

Here we describe a previously unidentified early, transient accumulation of neutrophils in the skin of HSV-1 infected mice that precedes a more robust, secondary wave of neutrophil influx later during infection^9^. The entry of neutrophils into both the skin and dLN was accompanied by Ly6C^hi^ monocytes, and agrees with a recent study that identified a transient, concomitant infiltration of both neutrophils and monocytes into skin wounds^14^.

It is well established that early innate immune responses are triggered by the activation of pathogen recognition receptors (PRRs) through detection of PAMPs in the case of microbial entry, or DAMPs during sterile injury, by tissue macrophages and mast cells, which leads to extravasation of neutrophils from the blood into the site of inflammation. HSV glycoproteins and viral genomic DNA have been reported to activate TLR2 and TLR9 respectively^15^,^16^,^17^. Similarly, damaged epidermal cells release DAMPs such as mitochondrial DNA that can induce neutrophil recruitment^18^.

We showed that immune sensing of viral-derived factors likely played only a minor role in the early influx of neutrophils, as evident after intradermal HSV-1 injection. On the other hand, sensing of tissue damage was likely the predominant cause of neutrophil recruitment to the skin and draining LN after HSV-1 infection following scarification, as there was no difference in the level of neutrophil recruitment between HSV- and saline-treated skin after scarification. This was reminiscent of the response that has been well characterised after sterile injury, where neutrophils are recruited and release factors to propagate further mononuclear cell recruitment^19^,^20^,^21^.

Neutrophils entering infected tissues are presented with ample opportunity for interactions with pathogens and infected cells. Previous studies have shown that neutrophils can modulate CD8^+^ T cell responses during lung influenza virus infection^8^, and that infected neutrophils may present antigen to effector CD8^+^ T cells in the lung^22^. Additionally, neutrophils have also been reported to act as antigen carriers after intradermal BCG and MVA (Modified Vaccinia Ankara) infections to the draining LN and bone marrow, respectively^4^,^23^. Our data using intravital microscopy suggested that neutrophils accumulated in the dermis just underneath the infected epidermal layer, indicating possible interactions with virus-infected keratinocytes. However, by tracking neutrophil migration from the skin, we found that only a very small number of neutrophils migrated to the draining LN. Although we labelled the skin at the early peak of neutrophil recruitment to mark the skin-infiltrating neutrophils, it remains possible that some migration occurs later in the response when neutrophil numbers increase again in the HSV lesion, after T cell priming and migration to the skin (days 5–7)^9^.

Hampton *et al*.^5^, using transgenic mice expressing photoconvertible KAEDE proteins, showed that following intradermal injection of heat-killed *S. aureus* bioparticles into the ear, a substantial number of neutrophils containing bacteria migrated from the injected skin to the draining LN. To directly compare with HSV-1 infection, we also infected mice intradermally in the flank skin with live *S. aureus* but observed very few migrated neutrophils. *S. aureus* employs a variety of immune evasion strategies that confer resistance to phagocytosis/killing by neutrophils, including the ability to secrete cytolytic toxins that kill leukocytes and innate immune cells^24^,^25^. Thus, injection of *S. aureus* bioparticles may induce a more sustained neutrophil response^5^. Additionally, differences in the site of bacterial injection (ear pinna vs. flank skin) might influence leukocyte recruitment and migration. We noted far fewer neutrophils infiltrating the flank skin compared to data published by other groups employing ear skin models^11^. Such differences have been attributed to the much thinner dermis of the ear skin^26^.

Interestingly, studies examining administration of DNA vaccine found that intradermal delivery into the ear pinna provided superior gene transfection and elicited stronger cell-mediated responses compared to administration into abdominal skin^27^,^28^. Additionally, a recent study found significantly higher numbers of mast cells in mouse ear skin than in back skin^29^. As mast cells may sense peptidoglycan from *S. aureus* via TLR2^30^ and are able to promote neutrophil recruitment^31^,^32^, the larger numbers of mast cells found in ear skin may explain differences. Furthermore, while Abadie *et al*.^4^ observed neutrophil migration to draining LN following inoculation of ear pinna with *M. bovis* BCG, another study utilising the same pathogen to infect the footpad failed to detect a similar skin-to-LN migration of neutrophils^33^. This suggests that inoculation of pathogens in distinct regions of skin may have variable immunological outcomes.

A number of studies have demonstrated an immunomodulatory role by neutrophils in both B and T cell responses during infection. Hampton *et al*.^5^ showed that neutrophils augmented both B and T cell proliferation, while implicating antigen shuttling and production of inflammatory cytokines as possible mechanisms that modulate lymphocyte expansion. Similarly, neutrophils were shown to directly interact with B cells in the LN and regulate antibody production^6^. Neutrophils can potentially also interact with antigen-presenting cells (APC) in the peripheral tissues. In one study employing *M. tuberculosis* infection, neutrophils facilitated early CD4^+^ T cell activation by helping non-infected DC to acquire antigen, as infected DCs themselves were impaired in migration^34^. Alternately, neutrophils may also control the magnitude of CD4^+^ T and B cell responses through immunosuppressive effects^35^.

In the case of epicutaneous HSV-1 infection, a previous study showed that neutrophil depletion did not alter the viral load or lesion severity throughout the course of infection^9^. Here we showed that ablation of neutrophils did not diminish nor enhance CD4^+^ or CD8^+^ T cell priming. The magnitude of expansion and degree of proliferation, as well as the expression of activation markers remained unchanged in neutrophil-depleted mice. This suggests that neutrophils did not significantly alter antigen presentation to T cells. Conversely, Tate *et al*.^8^ reported that while neutrophil depletion during influenza virus infection did not affect antigen presentation to CD8^+^ T cells, they observed diminished CD8^+^ T cell expansion as well as impaired antiviral cytokine expression.

Finally, neutrophils have been implicated in influencing migration of effector CD8^+^ T cells to peripheral tissues, including the skin^36^,^37^. More recently, Lim *et al*.^7^ showed that neutrophils leave behind CXCL12-enriched migratory trails in influenza-infected trachea, which in turn promoted the migration and infiltration of effector CD8^+^ T cells. Following neutrophil depletion, we found no significant difference in the numbers of both effector CD4^+^ and CD8^+^ T cells recruited to HSV-infected flank skin. During mucosal HSV-2 infection, it has been shown that CD4^+^ T cells infiltrate infected tissues and promote local CXCL9 and CXCL10 production, which in turn facilitates CD8^+^ T cell accumulation, demonstrating distinct stages of CD4^+^ and CD8^+^ T cell recruitment to the infected tissues^38^. Similarly, we have previously shown that effector CD4^+^ T cells arrived at HSV-1 infected skin ~1 day earlier than CD8^+^ T cells^13^. Thus, with multiple chemokine pathways regulating effector T cell recruitment, it was possible that in the case of HSV-1 the presence of other chemokine cues promoted by these early-infiltrating CD4^+^ T cells, such as CXCL9 and CXCL10, could have compensated for neutrophil-dependent chemokinetic signals due to the late entry of CD8^+^ T cells into the effector sites.

Our results show that despite recruitment of neutrophils to the skin in response to live viral or bacterial infection, neutrophils recruited to the skin do not migrate extensively to draining lymph nodes. Moreover, neutrophils were not required for T cell priming and recruitment to infected skin after HSV-1 infection. These data suggest that the pathogen and route of infection may dictate the functional contribution of neutrophils towards adaptive immunity.

## Methods

### Mice and Infections

C57BL/6, gBT-I x B6.SJL-*Ptprc*^*a*^*Pep3*^*b*^/BoyJ (gBT-I.CD45.1), gDT-II x B6.CD45.1 mice were bred in the Department of Microbiology and Immunology. gBT-I and gDT-II encode transgenes expressing T cell receptor recognising the HSV-1 glycoprotein B-derived epitope gB_498–505_ and glycoprotein D-derived epitope gD_315–327_, respectively. LysM-EGFP mice were provided by M. Hickey (Monash University). Animal experiments were approved by The University of Melbourne Animal Ethics Committee. All methods were carried out in accordance with guidelines and regulations of the University of Melbourne.

### TRITC painting

Tetramethylrhodamine-5-isothiocyanate (TRITC; Life Technologies) was dissolved in DMSO and diluted to 0.5% (v/v) in acetone. The TRITC solution was painted on the scarified skin region (1 cm^2^ diameter) of anaesthetised mice (6–7 hr after scarification or infection) in a 10 μl volume and allowed to dry. The scar was then re-hydrated with 10 μl PBS and re-bandaged as described in ref. 39.

### HSV and *S. aureus* infections

Epicutaneous infection with HSV-1 KOS was performed as described elsewhere^39^. Briefly, mice were anaesthetised with 1:1 mixture of ketamine and Xylazil (10 μl/g of body weight) by intraperitoneal injection. Hair was removed from the flank and depilated (Veet, Reckitt Benckiser). Scarification was performed by lightly abrading the flank skin and infected with 10^6^ plaque-forming units (pfu) of HSV-1.

For bacterial infection, *S. aureus* JKD6009 GFP^40^ was grown in brain heart infusion (BHI) broth at 37 °C to mid-log phase, and the concentration determined through optical density measurement at 600 nm (OD_600_) using a spectrophotometer. Inoculum was also plated on HI-CM plates to verify the cfu on the following day.

For intradermal infections, mice were anaesthetised with Ketamine/Xylazil as described above. For HSV-1, virus was diluted to 10^6^ or 10^7 ^pfu/20 μl in PBS. For *S. aureus*, bacteria was diluted to 10^6^ or 10^7^ colony-forming units (cfu)/20 μl in PBS with concentration determined using spectrophotometer. Virus or bacteria were intradermally injected into skin covering the tip of the spleen with a 29½G insulin syringe.

### Cell isolation and flow cytometry

Mice were sacrificed by CO_2_ euthanasia. For experiments involving detection of skin neutrophils, 1 × 1 cm^2^ skins containing the infection site were excised using a scalpel blade. For experiments examining skin T cells, 2 strips of 0.5 × 1.5 cm^2^ skins containing the herpes lesions were excised. Skin samples were placed into 1 ml collagenase type III (3 mg/ml in RPMI-5), finely chopped and incubated at 37 °C for 90 min, prior to transferring into 5 ml RPMI-10 and then vigorously mixed. Cell debris was filtered out with a 70 μm sieve. After resuspension in 1 ml FACS buffer (PBS with 2% FCS, 5 mM EDTA, 0.1% NaN_3_), cells were filtered again with a 30 μm sieve prior to antibody staining.

Lymph nodes and/or spleens were harvested and pushed through a 70 μm sieve to obtain single cell suspensions, and resuspended in FACS buffer for antibody staining. Propidium iodide was added to samples prior to acquisition by flow cytometry (BD FACS Canto or BD Fortessa). Data were analysed using FlowJo software (TreeStar).

The following antibodies/reagents were purchased from BD Pharmingen: CD11b-APC-Cy7 (M1/70), CD19-PerCP-Cy5.5 (1D3), NK1.1-PerCP-Cy5.5 (PK136), Ly6G-PE (1A8), Ly6C-FITC (AL-21), Ly6C and Ly6G (Gr-1)-APC (RB6-8C5), CD69-PE-Cy7 (H1.2F3), Vα2-PE-Cy7 (B20.1); eBioscience: CD11c-PE-Cy7 (N418), CD3e-PerCP-Cy5.5 (145-2C11), MHC II (I-A/I-E)-Alexa Fluor 700 (M5/114.15.2), CD45.1-APC (A20), CD44-Alexa Fluor 700 (IM7), CD8α-APC-eFluor780 (53-6.7), CD62L-PerCP-Cy5.5 (MEL-14); BioLegend: CD4-Brilliant Violet 605 (RM4-5), Vα3.2-PE (RR3-16).

### *In vivo* depletion of neutrophils

Mice were injected i.p. with 500 μg anti-Ly6G (1A8, Bio X Cell) depleting antibodies in 200 μl PBS at indicated time points. Depletion efficiency was determined at experiment endpoint by co-staining for anti-Ly6C and anti-Gr-1 to detect neutrophils (Ly6C^int^ Gr-1^hi^).

### T cell isolation, labelling and adoptive transfer

T cells were enriched from naïve lymph nodes and/or spleens of female transgenic mice through negative enrichment of CD4^+^ or CD8^+^ T cells, for gDT-II and gBT-I, respectively. For gDT-II cells, further positive magnetic enrichment was performed as described elsewhere^41^. Purities of 50–60% for gDT-II and >90% for gBT-I were attained. For experiments involving dye labelling of T cells, purified cells were incubated with 5 μM CellTrace Violet at 37 °C for 10 min, and washed in complete media. T cells were then adoptively transferred intravenously into recipient mice prior to infection.

### Immunofluorescence microscopy

Lymph nodes were harvested and fixed in paraformaldehyde-lysine-periodate (PLP) fixative (0.05 M phosphate buffer added with 0.1 M L-lysine (pH 7.4; Sigma-Aldrich), 2 mg/ml NaIO_4_ (BDH Chemicals) and 4% (v/v) paraformaldehyde (Electron Microscopy Sciences, ProSciTech)) for 6–8 hrs, washed twice in PBS for 10 min and incubated in 20% sucrose overnight at 4 °C. Fixed tissues were then embedded in OCT compound (Tissue Tek, Sakura Finetek) and snap frozen in liquid nitrogen, prior to storage at −80 °C. For cryosectioning, tissues sections were cut at 12 μm thickness with a cryostat (Leica CM3050S) and air-dried. Sections were then fixed in acetone for 5 min, dried and then blocked for 20 min (Protein Block X0909, DAKO) at room temperature (RT), before staining with primary antibodies (Pacific Blue anti-B220 (RA3-6B2, BioLegend), purified polyclonal anti-LYVE1 (Abcam), Alexa Fluor 488 anti-PNAd (MECA-79, eBioscience), Alexa Fluor 647 anti-Ly6G (1A8, BioLegend)) at RT for 1.5 hr, washed twice in PBS for 10 min and further stained with secondary antibodies (donkey anti-rabbit Alexa Fluor 568 (Life Technologies)) for 30 min. Sections were mounted using Prolong Gold antifade reagent (Life Technologies) on glass slides, and images acquired using an LSM710 confocal microscope (Carl Zeiss). Post-acquisition processing was performed using Imaris (Bitplane), ImageJ (NIH) and Photoshop (Adobe).

### Intravital skin imaging

Mice were anaesthetised with isoflurane (Cenvet; 2.5% for induction, 1–1.5% for maintenance, vaporised at 80:20 mixture of O_2_ and air), and were shaved on the left flank and hair depilated for flank skin imaging as described elsewhere^42^. Briefly, two incisions (~15 mm apart) were made longitudinally along the left flank, cutting through the dermis, and the peritoneum separated by cutting away the connective tissues underneath the skin. A 18 mm-wide x 1 mm thick stainless steel platform was inserted under the exposed dermis, which was glued upon the platform with Vetbond tissue adhesive (3 M). The edges of the skin were lined with vacuum grease (Dow Corning), upon which a glass coverslip was placed. Imaging was performed with an upright LSM710 NLO multiphoton microscope (Carl Zeiss) with a 20x/1.0 NA water immersion objective enclosed in an environmental chamber (Precision Plastics) that was maintained at 35 °C with heated air. Fluorescence excitation was provided by a Chameleon Vision II Ti:sapphire laser (Coherent) with dispersion correction and fluorescence emission detected using external non-descanned photomultiplier tubes. EGFP and second-harmonic generation were excited at 920 nm. GFP-hi cells were motile neutrophils and GFP-lo cells were macrophages/monocytes. For four-dimensional data sets, three-dimensional stacks were captured every 60 seconds for at least 30 min. Raw imaging data were then processed with Imaris 7 (Bitplane) and drift corrected, and movies generated in Imaris and composed in After Effects (Adobe).

## Additional Information

**How to cite this article:** Hor, J. L. *et al*. Neutrophils are dispensable in the modulation of T cell immunity against cutaneous HSV-1 infection. *Sci. Rep.* **7**, 41091; doi: 10.1038/srep41091 (2017).

**Publisher's note:** Springer Nature remains neutral with regard to jurisdictional claims in published maps and institutional affiliations.

## Supplementary Material

Supplemental Figures
