# Supplementary material for: Neutrophils are dispensable in the modulation of T cell immunity against cutaneous HSV-1 infection
Source: Sci Rep. 2017 Jan 23;7:41091. doi: 10.1038/srep41091 (PMC5253768; doi:10.1038/srep41091)
Supplement: Supplemental Figures [file srep41091-s1.pdf]

Neutrophils are dispensable in the modulation of T cell immunity against cutaneous HSV-1 infection

Jyh Liang Hor[1,2], William R. Heath[1,2] and Scott N. Mueller[1,2,*]

[1]Department of Microbiology and Immunology, The University of Melbourne, at the Peter Doherty Institute for Infection and Immunity, Melbourne 3000, Australia

[2]The Australian Research Council Centre of Excellence in Advanced Molecular Imaging, The University of Melbourne, Melbourne 3000, Australia

Correspondence: smue@unimelb.edu.au

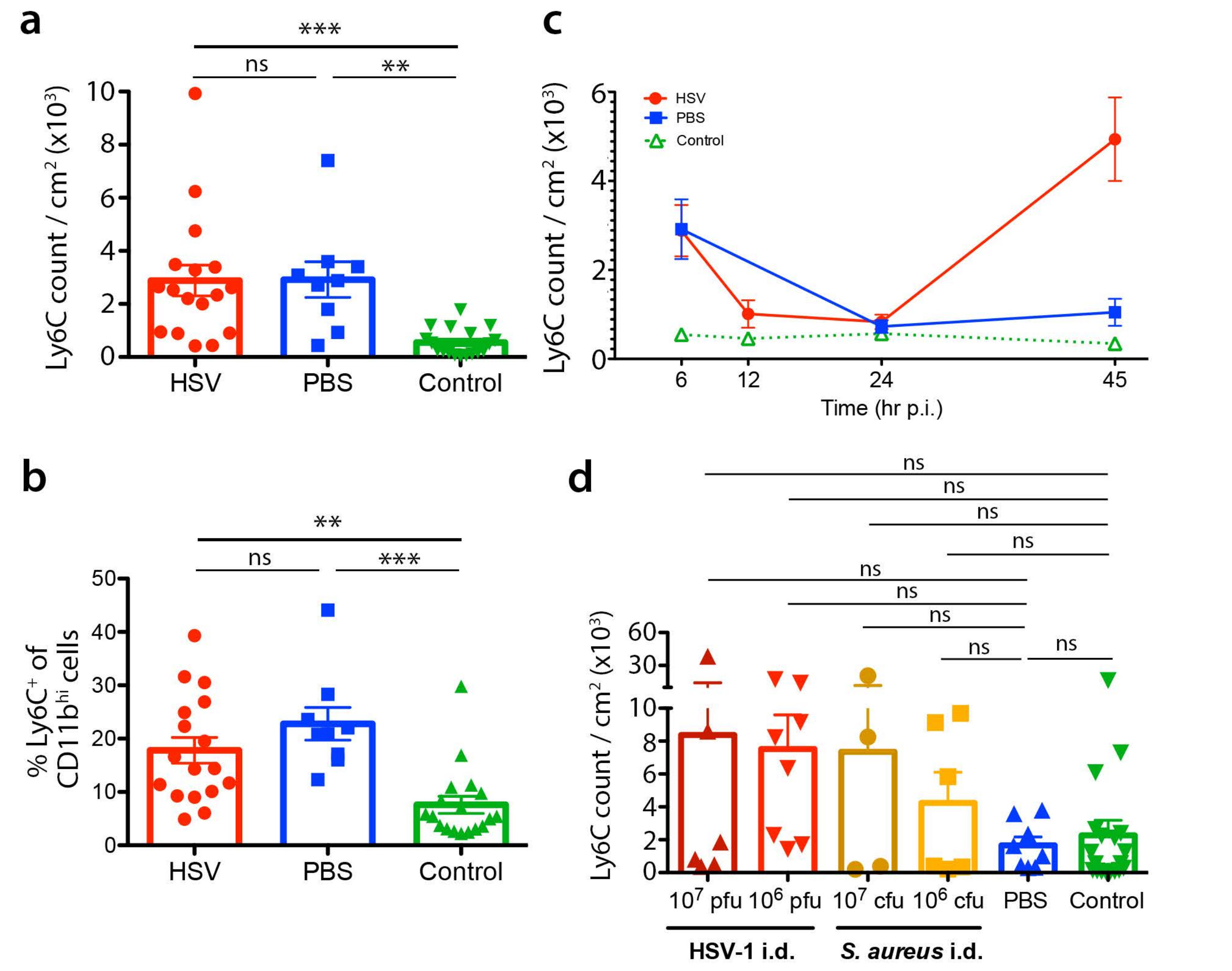

Figure S1. Monocyte recruitment to the skin after HSV-1 infection.
C57BL/6 mice were dermally scarified and treated as described in Fig. 1a.
(a) Total number of Ly6C^{hi} monocytes recovered from 1cm^2 skin at 6 hr post-scarification.
(b) Proportion of Ly6C^{hi} monocytes amongst CD11b$^+$ population at 6 hr post-scarification.
(c) Time course depicting total number of Ly6C^{hi} monocytes population from 6 hr to 45 hr post-scarification.
(a-c) Data pooled from 2-5 independent experiments, n=9-18 mice per group (a-b), n=5-18 mice per group (c).
(d) C57BL/6 mice were intradermally injected with HSV-1 (10^7 pfu, dark red or 10^6 pfu, red), *S. aureus* (10^7 cfu, dark orange or 10^6 cfu, orange), PBS (blue) or left untreated (green), as described in Fig. 1e. Shown are total numbers of Ly6G^{hi} neutrophils recovered from skin excised at 6 hr p.i. Data pooled from 2 independent experiments, n=4-8 per group, n=19 for control group.
(a-d) Error bars represent mean ± SEM. **$p<0.01$, ***$p<0.001$, one-way ANOVA, Tukey's multiple comparisons. ns, not significant.

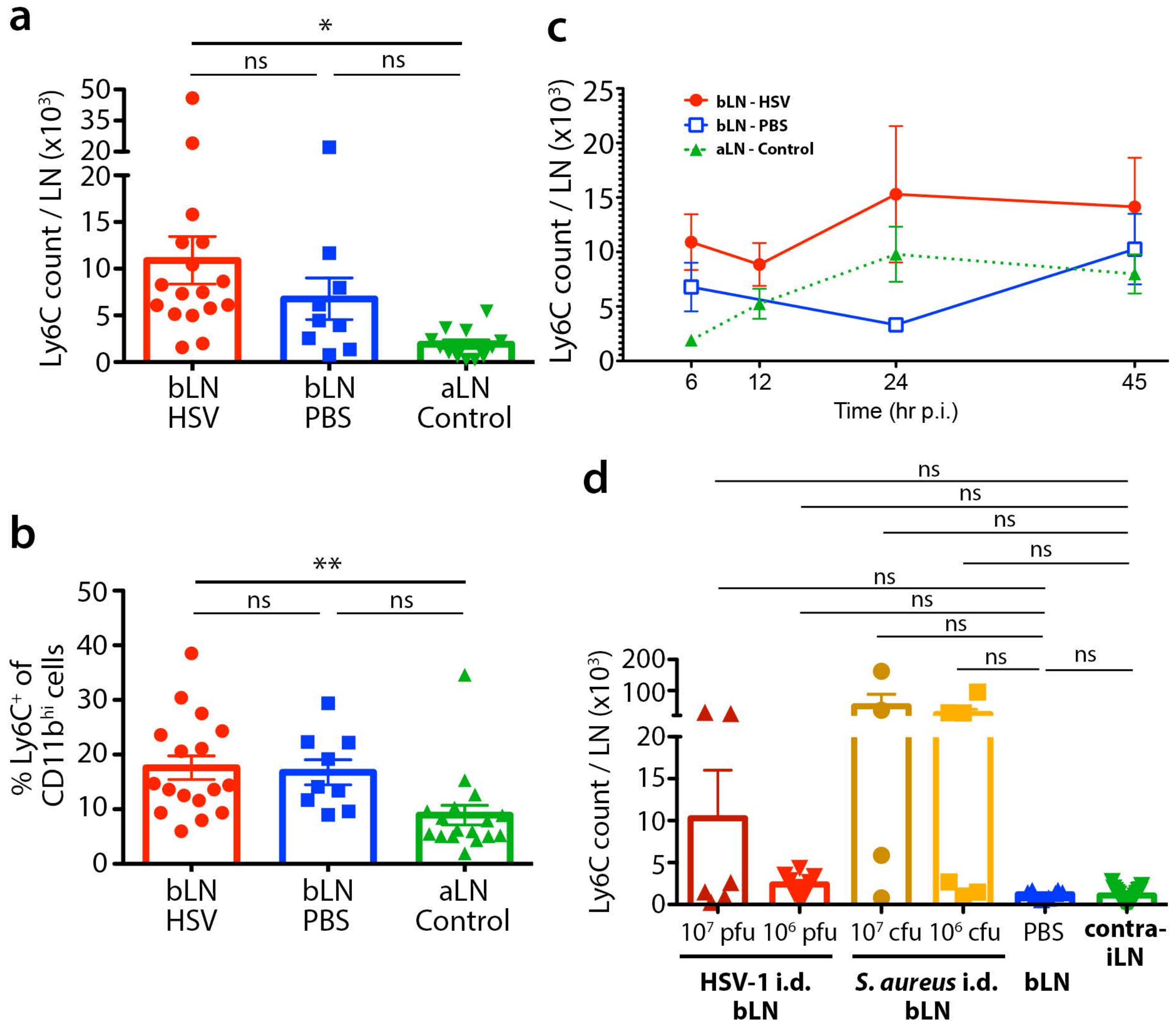

Figure S2. Monocyte recruitment to the draining bLN after HSV-1 infection.
C57BL/6 mice were dermally scarified and treated as described in Fig. 1a.
(a) Total number of Ly6C^{hi} monocytes recovered from bLN at 6 hr post-scarification.
(b) Proportion of Ly6C^{hi} monocytes amongst CD11b$^+$ population at 6 hr post-scarification.
(c) Time course depicting total number of Ly6C^{hi} monocytes population from 6 hr to 45 hr post-scarification.
(a-c) Data pooled from 2-5 independent experiments, n=9-18 mice per group (a-b), n=5-18 mice per group (d).
(d) C57BL/6 mice were intradermally injected with HSV-1 (10^7 pfu, dark red or 10^6 pfu, red), *S. aureus* (10^7 cfu, dark orange or 10^6 cfu, orange), PBS (blue) or left untreated (green), as described in Fig. 1e. Shown are total numbers of Ly6G^{hi} neutrophils recovered from bLN or contralateral-inguinal LN containing the injection site excised at 6 hr p.i. Data pooled from 2 independent experiments, n=4-8 per group, n=19 for control group.
(a-d) Error bars represent mean ± SEM. **p<0.01, ***p<0.001, one-way ANOVA, Tukey's multiple comparisons. ns, not significant.

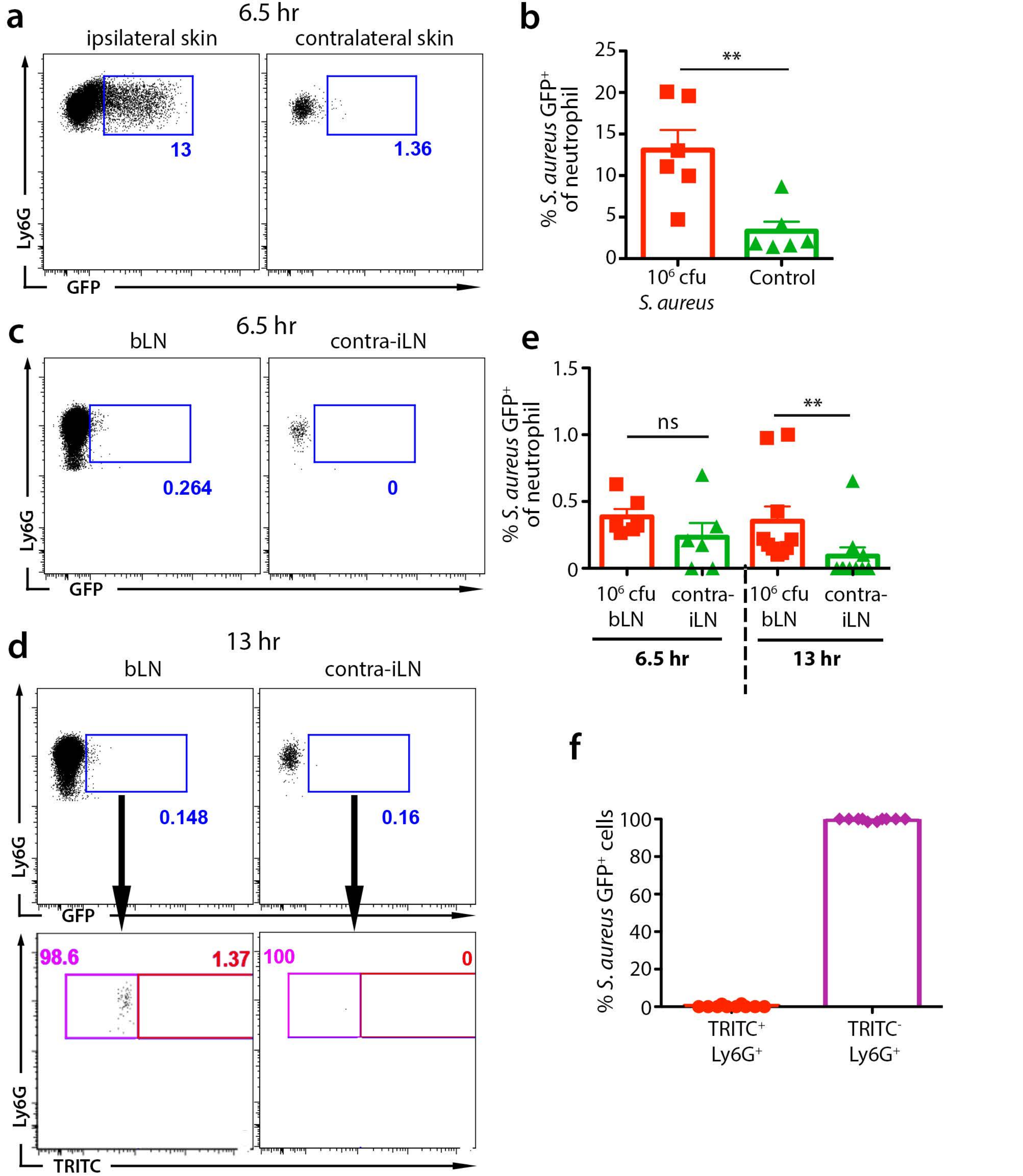

Figure S3 Minimal migration of S. aureus-containing neutrophils into draining LN. C57BL/6 mice were intradermally injected with 10^6 cfu GFP-expressing *S. aureus*. 1cm^2 skin containing the injection site as well as the draining bLN were excised at 6.5 hr p.i. for flow cytometry analysis. Some mice were painted with TRITC at 6.5 hr p.i. and draining bLN harvested at 13 hr p.i. for analysis.

(a) Representative dot plots showing gating of GFP$^+$ Ly6G^{hi} neutrophils in the skin (infected, left; control, right) at 6.5 hr p.i.

(b) Proportion of *S. aureus* GFP$^+$ cells of all neutrophils per cm^2 skin at 6.5 hr p.i.

(c, d) Representative dot plots showing gating of GFP$^+$ Ly6G^{hi} neutrophils in the LN (draining bLN, left; non-draining iLN, right) at 6.5 hr (c) and 13 hr p.i. (d). Bottom panels of (d) showed further gating of GFP$^+$ Ly6G^{hi} cells into TRITC$^+$ (red) and TRITC$^-$ (magenta) populations.

(e) Proportion of *S. aureus* GFP$^+$ cells of all neutrophils per LN at 6.5 hr and 13 hr p.i.

(f) Proportion of TRITC$^+$ or TRITC$^-$ GFP$^+$ Ly6G^{hi} neutrophils per LN at 13 hr p.i.

Data pooled from 2 independent experiments, n=6-10 mice per group. Error bars represent mean ± SEM. **p<0.01, Mann Whitney *U* test. ns, not significant.

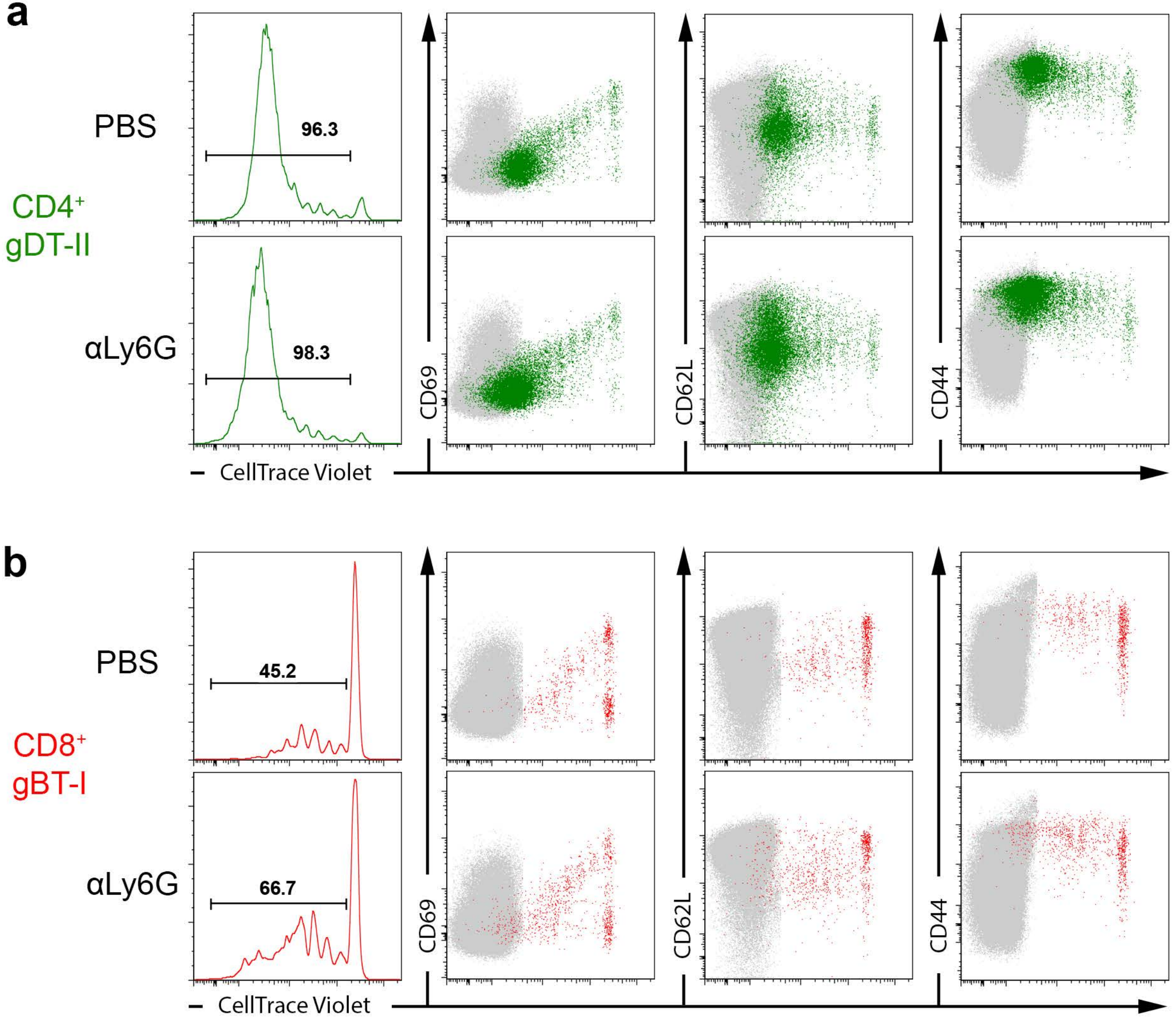

Figure S4. Expression of activation markers in HSV-specific CD4⁺ and CD8⁺ T cells after neutrophil depletion.

(a, b) Representative dot plots depicting the division and expression of activation markers CD69, CD62L and CD44 in CD4⁺ gDT-II (a) and CD8⁺ gBT-I (b) T cells between non-depleted (top row) and anti-Ly6G depleted (bottom row) groups at Day 3 p.i. corresponding to Fig. 4. Data representative of 3 independent experiments.

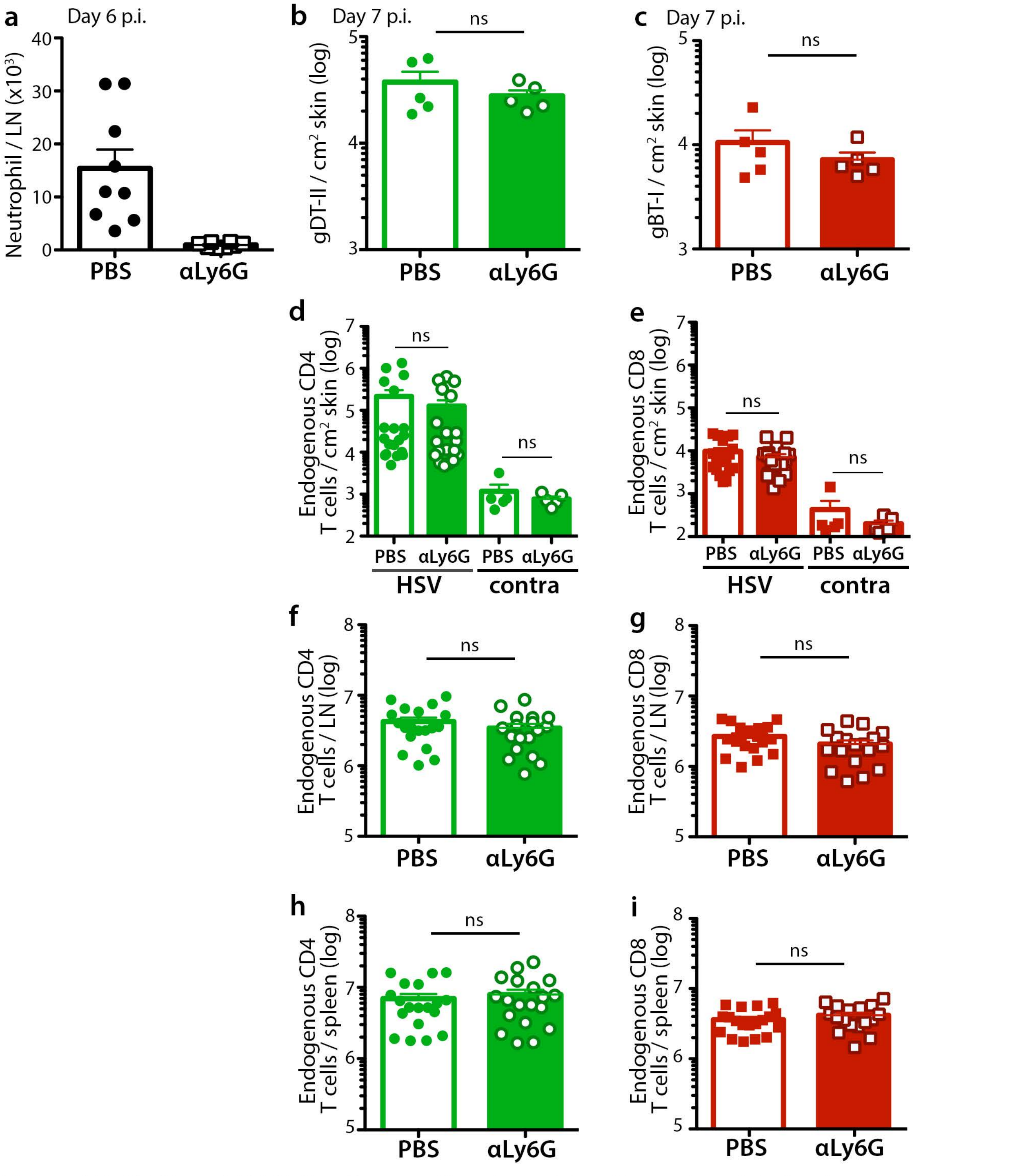

Figure S5. $CD4^+$ and $CD8^+$ T cell migration into skin at Day 7 post-infection.
C57BL/6 mice were adoptively transferred with T cells and neutrophils depleted with anti-Ly6G as described in Fig. 5a.
(a) Total number of neutrophils recovered from draining bLN in both non-depleted (open bars) and anti-Ly6G depleted (filled bars) mice at Day 6 p.i. Error bars represent mean ± SEM. Data pooled from 2 independent experiments.
(b, c) Total number of $CD4^+$ gDT-II (b) and $CD8^+$ gBT-I (c) T cells per cm^2 of excised Day 7 p.i. skin in non-depleted (open bars) and anti-Ly6G depleted (filled bars) groups. Data from 1 experiment, n=5 mice per group. Error bars represent mean ± SEM. Unpaired Student t test on log transformed values. ns, not significant.
(d, e) Total number of endogenous $CD4^+$ (d) and $CD8^+$ (e) T cells per cm^2 of excised Day 6 p.i. skin in non-depleted (open bars) and anti-Ly6G depleted (filled bars) groups.
(f-i) Total number of endogenous $CD4^+$ (f, h) and $CD8^+$ (g, i) T cells per bLN (e, f) and spleen (h, i) in non-depleted (open bars) and anti-Ly6G depleted (filled bars) groups.
(d-i) Data pooled from 4 independent experiments, n=18-19 mice per group. Error bars represent mean ± SEM. One-way ANOVA, Tukey's multiple comparisons on log transformed values (d, e) or unpaired Student t tests (f-i). ns, not significant.
